# Choosing Between Fixed and Removable Prosthetic Modalities for Completely Edentulous Patients: A Systematic Review of Evidence-Based Outcomes

**DOI:** 10.7759/cureus.101213

**Published:** 2026-01-10

**Authors:** Marwa Abdelrahman Ali Hassan, Ramah Mohammed Elhadi, Manahel Osman, Safaa Ali Mohammed Abdalrahman, Marwa Abdelhay Elzibair Eltahir, Salma Nureldinn

**Affiliations:** 1 Dentistry, Safwa Al Muhaidib Dental Center, Makkah, SAU; 2 Dentistry, Al Razi University, Buraydah, SAU; 3 Oral and Maxillofacial Surgery, Salisbury District Hospital, Salisbury, GBR; 4 Oral and Maxillofacial Surgery, Dudley Group NHS Foundation Trust, Dudley, GBR; 5 Dentistry, Private Practice, Sheffield, GBR; 6 Dentistry, Khartoum University, Khartoum, SDN

**Keywords:** complete edentulism, fixed dental prosthesis, implant-supported prosthesis, oral health-related quality of life, patient-reported outcomes, removable overdenture

## Abstract

Complete edentulism significantly impacts masticatory function, aesthetics, and quality of life. The selection between fixed and removable prosthetic modalities is a critical clinical decision. This systematic review aims to compare the clinical and patient-reported outcomes of fixed versus removable prosthetic rehabilitations for completely edentulous patients.

A systematic search of PubMed/MEDLINE, Scopus, Embase, and Web of Science databases was conducted up to December 2025. Studies were selected based on predefined PICOS criteria, including randomized controlled trials and observational studies. Risk of bias was assessed using the Cochrane RoB 2 tool and the Newcastle-Ottawa Scale. A narrative synthesis was performed due to significant heterogeneity. Ten studies were included. Fixed implant-supported prostheses demonstrated high long-term survival rates and superior masticatory efficiency and occlusal stability compared to removable options. Patient-reported outcomes, particularly oral health-related quality of life, were significantly better with any implant-supported prosthesis (fixed or removable) than with conventional complete dentures. Fixed prostheses (FPs) were associated with technical complications, while removable overdentures (ODs) presented challenges with occlusal wear. Conventional denture satisfaction was primarily linked to retention, comfort, and aesthetic quality. Implant-supported prostheses offer substantially better clinical and patient-reported outcomes than conventional dentures for completely edentulous patients. The choice between a fixed and removable modality involves a trade-off: FPs provide superior function and stability but require more complex maintenance, whereas removable ODs offer easier hygiene and a favorable balance of benefits. Treatment must be individualized based on anatomical factors, patient priorities, and clinical feasibility.

## Introduction and background

Complete edentulism remains a common oral health condition worldwide and continues to pose a substantial clinical and public health challenge, particularly among aging populations. It is associated with impaired masticatory efficiency, compromised esthetics and speech, nutritional limitations, and reduced oral health-related quality of life (OHRQoL) [1]. As life expectancy increases and expectations for oral function and comfort rise, the demand for predictable and patient-centered prosthodontic rehabilitation in completely edentulous individuals has grown accordingly.

A central clinical decision in the management of complete edentulism is the choice between fixed and removable prosthetic rehabilitation [2]. Fixed prosthetic options, most commonly implant-supported fixed complete dentures (CDs), are designed to provide high stability, improved chewing efficiency, and a sensation closer to natural dentition. In contrast, removable prostheses, including conventional CDs and implant-retained overdentures (ODs), remain widely used due to their relative affordability, simpler fabrication, and ease of hygiene maintenance [3]. For readers less familiar with implant prosthodontics, this distinction represents not only a difference in prosthesis design but also a difference in surgical requirements, maintenance demands, and long-term patient commitment.

The selection of an appropriate prosthetic modality is multifactorial and extends beyond prosthetic preference alone. Key clinical decision points include residual ridge anatomy, available bone volume and quality, systemic health status, and the patient’s ability to maintain oral hygiene, particularly in elderly or medically compromised individuals [4]. Financial considerations and health-system constraints also play a decisive role, as fixed implant-supported solutions often involve higher initial costs and greater surgical complexity compared with removable alternatives. In addition, patient-related and caregiver-related factors, such as expectations, manual dexterity, motivation, and psychosocial support, can significantly influence adaptation, satisfaction, and long-term success, especially in removable denture therapy [5].

Despite continuous advances in implant materials, surgical protocols, and prosthetic designs, the evidence comparing fixed and removable prosthetic modalities remains heterogeneous. Reported outcomes vary across studies, with inconsistencies in implant survival rates, prosthesis longevity, masticatory performance, patient satisfaction, and OHRQoL measures [6]. This variability complicates clinical decision-making, particularly for non-specialists seeking clear, evidence-based guidance for treatment planning in completely edentulous patients.

Given these considerations, a systematic synthesis of the available evidence is warranted. The present systematic review aims to critically compare fixed and removable prosthetic modalities for completely edentulous patients by evaluating both clinical outcomes and patient-reported measures. By consolidating and appraising current evidence, this review seeks to support informed, individualized, and evidence-based prosthodontic decision-making in clinical practice.

## Review

Methodology

Study Design and Protocol

This systematic review was conducted in accordance with the Preferred Reporting Items for Systematic Reviews and Meta-Analyses (PRISMA) guidelines to ensure transparency, reproducibility, and methodological rigor [7]. A predefined protocol was developed prior to the commencement of the review, detailing the objectives, eligibility criteria, search strategy, and planned outcomes.

Eligibility Criteria

Studies were selected based on the PICOS framework. Population included completely edentulous patients requiring prosthetic rehabilitation. Interventions comprised fixed prosthetic modalities, including implant-supported fixed dentures, while comparators included removable prosthetic modalities, such as conventional CDs or implant-retained ODs. Outcomes of interest were both clinical and patient-reported, including implant survival rate, prosthesis survival, masticatory efficiency, oral health-related quality of life, and patient satisfaction. Study designs included randomized controlled trials, controlled clinical trials, and observational studies published in English. Studies were excluded if they involved partial edentulism, case reports, reviews, or animal studies.

Information Sources and Search Strategy

A comprehensive literature search was conducted in PubMed, Scopus, Web of Science, and Embase databases, covering all studies published up to December 2025. The search strategy combined Medical Subject Headings (MeSH) and free-text terms related to “edentulism,” “fixed prosthesis,” “removable prosthesis,” “implant-supported dentures,” and “overdentures.” Boolean operators were applied to refine the search, ensuring a high sensitivity for relevant studies. Reference lists of included studies were also screened to identify additional eligible studies. The detailed search strings for the databases are provided in the Appendices section.

Study Selection

All retrieved records were imported into EndNote 21 (Clarivate, Philadelphia, USA), and duplicates were identified and removed systematically. Two independent reviewers screened titles and abstracts for relevance, followed by full-text review of potentially eligible studies. Discrepancies between reviewers were resolved through discussion or consultation with a third reviewer to ensure accuracy and minimize selection bias.

Data Extraction

Data from included studies were extracted independently by two reviewers using a standardized data collection form. Extracted information included study characteristics (country, study design, sample size, patient demographics), type of prosthesis, follow-up duration, primary outcomes, and comparator interventions. Any disagreements were resolved by consensus.

Risk of Bias Assessment

The methodological quality of included studies was assessed independently by two reviewers. Randomized controlled trials were evaluated using the Cochrane Risk of Bias 2 (RoB 2) tool [8], while observational studies were assessed using the Newcastle-Ottawa Scale (NOS) [9]. Studies were categorized as low, moderate, or high risk of bias, and results were incorporated into the interpretation of findings.

Data Synthesis

Given the substantial heterogeneity among included studies with respect to prosthetic designs, follow-up durations, outcome measures, and assessment methods, a meta-analysis was not feasible. The variability in clinical protocols, patient populations, and outcome reporting precluded meaningful quantitative synthesis. Therefore, a narrative synthesis of results was performed, highlighting patterns and differences between fixed and removable prosthetic modalities, emphasizing both clinical outcomes and patient-reported measures.

Results

Study Selection Process

**Figure 1:**
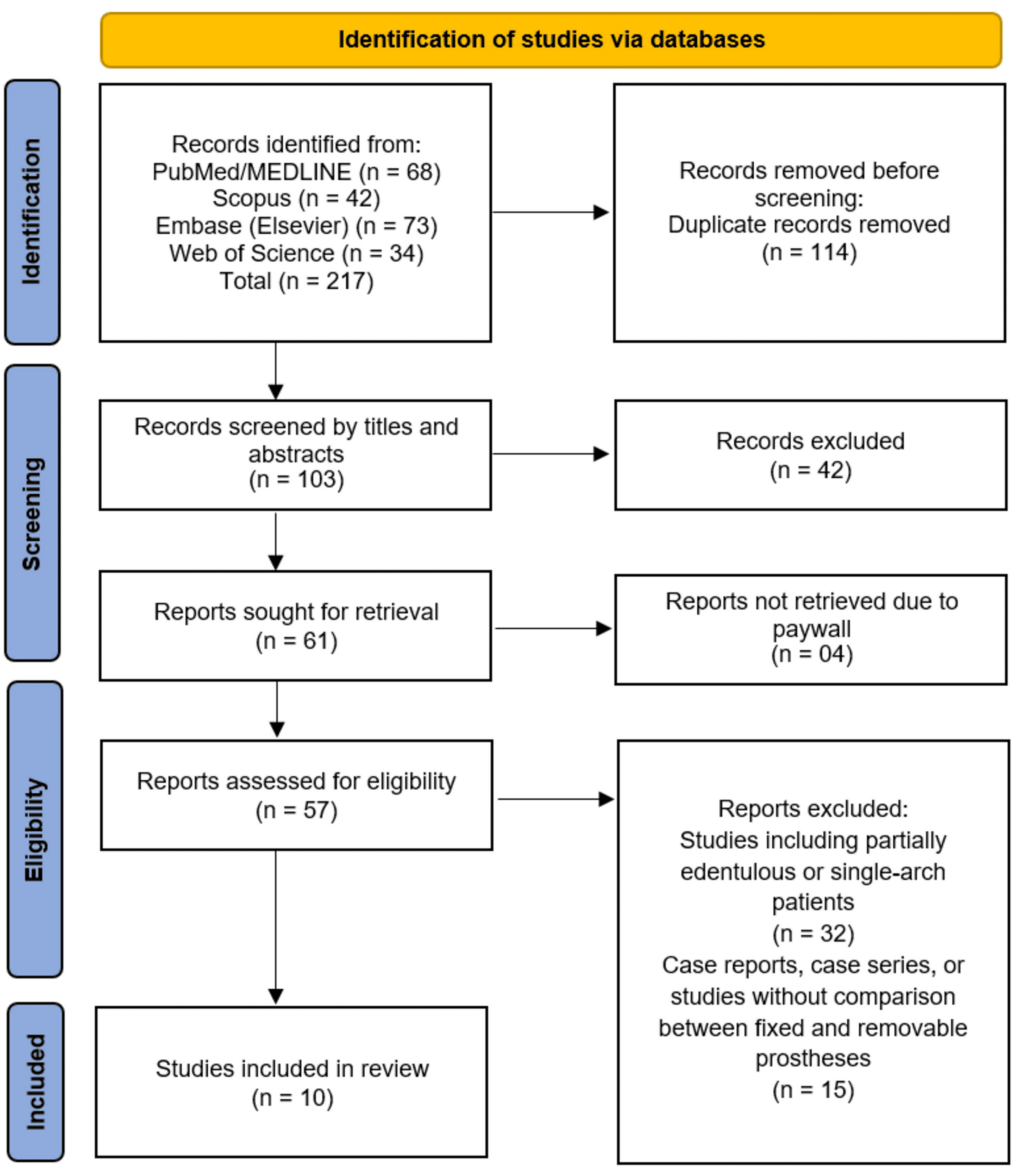
PRISMA Flow Diagram PRISMA: Preferred Reporting Items for Systematic Reviews and Meta-Analyses

The systematic search and study selection process are summarized in the PRISMA flow diagram (Figure 1). A total of 217 records were initially identified through comprehensive database searches of PubMed/MEDLINE (n=68), Scopus (n=42), Embase (n=73), and Web of Science (n=34). After the removal of 114 duplicate records, the titles and abstracts of 103 unique records were screened for relevance. Following this initial screening, 42 records were excluded, leaving 61 full-text articles sought for retrieval. Four reports could not be retrieved due to paywall restrictions. The remaining 57 full-text articles were assessed for eligibility against the inclusion criteria. A significant number of studies (n=32) were excluded for including partially edentulous or single-arch patient populations, which did not align with the review's focus on completely edentulous patients. A further 15 reports were excluded as they were case reports, case series, or lacked a direct comparative analysis between fixed and removable prosthetic modalities. Ultimately, 10 studies [10-19] met all inclusion criteria and were incorporated into the qualitative synthesis of this systematic review.

Study Selection and Characteristics

A total of 10 studies were included in this systematic review, comprising a range of research designs and geographical contexts. The characteristics of these studies are summarized in Table 1. The included research featured randomized controlled trials (RCTs) [10,12], retrospective cohort or observational studies [11,13-15], prospective observational studies [15,19], and cross-sectional or survey-based investigations [16-18]. Sample sizes varied from 20 participants [10] to 432 enrolled patients [17]. The follow-up durations ranged from three months [12] to a mean of 52 months [14], with one study reporting outcomes up to six years [13]. The studies originated from multiple countries, including Egypt, China, Italy, Spain, Japan, Croatia, and Brazil, and primarily focused on comparing outcomes between fixed implant-supported prostheses, removable implant-supported ODs, and conventional CDs.

**Table 1:** Characteristics of Included Studies Evaluating Fixed and Removable Prosthetic Modalities in Completely Edentulous Patients RCT: randomized controlled trial; NR: not reported; 3D: three-dimensional; EMG: electromyography; OHIP: oral health impact profile; OHIP-14: 14-item oral health impact profile; OHIP-19: 19-item oral health impact profile; OHRQoL: oral health-related quality of life; DIP: denture impact profile; CDs: complete dentures; FP: fixed prosthesis; OD: overdenture; M/F: male/female; All-on-Four: full-arch implant rehabilitation concept using four implants.

Author (Year)	Country	Study Design	Sample Size (n)	Patient Characteristics (Age, Sex)	Type of Prosthesis	Follow-up Duration	Comparator	Primary Outcomes Assessed
Ali et al., [10] (2025)	Egypt	RCT	20	Adults, mixed sex	Mandibular implant overdentures (conventional vs 3D-printed)	12 months	Conventional	Occlusal wear, Occlusal force distribution
Yan et al., [11] (2025)	China	Retrospective 3D analysis	22	Edentulous	Implant-supported fixed (maxilla)	NR	None	Incisor position; residual bone dimensions; crown-bone angles
Elboraey et al., [12] (2024)	Egypt	Randomized clinical trial	22	Completely edentulous mandibular patients	Removable mandibular implant-supported overdenture; Implant-retained fixed mandibular prosthesis	3 months	Removable vs fixed mandibular implant-retained prostheses	Occlusal equilibration, muscle activity (EMG), oral health–related quality of life (OHIP-19)
Nagni et al., [13] (2023)	Italy	Retrospective study	30	Sex: M/F	Fixed full-arch implant prosthesis (4 or 6 implants)	Up to 6 years	4 implants (All-on-Four) vs 6 implants	Implant survival, bone loss, peri-implant health, complications, satisfaction
Sánchez-Torres et al., [14] (2021)	Spain	Retrospective cohort study	56 patients (72 arches)	Mean age: 64 ± 11.1 years; 26 men, 30 women	Immediately loaded complete-arch implant-supported prostheses	Mean 52 ± 26 months	Patients with vs without mechanical complications	Mechanical complications, patient satisfaction, oral health–related quality of life (OHIP-14)
Di et al., [15] (2013)	China	Prospective observational study (convenience sample)	69	Mean age: 56.7 years; 37 men, 32 women	Fixed full-arch implant-supported prosthesis (All-on-Four concept)	Mean 33.7 months (range: 12–56 months)	Mandible vs maxilla; fresh extraction vs healed sites	Implant survival rate, marginal bone loss, complications, patient-reported satisfaction
Martín‐Ares et al., [16] (2016)	Spain	Comparative cross-sectional study	150	Geriatric patients	CD; implant-supported FP; implant-supported OD	NR	CD vs FP vs OD	Patient satisfaction, functional satisfaction, oral hygiene maintenance, OHRQoL (OHIP, DIP)
Yamaga et al., [17] (2019)	Japan	Observational study (registry-based analysis)	432 enrolled; 267 completed	NR	Complete dentures	Usage period assessed	None	OHRQoL
Čelebić et al., [18] (2003)	Croatia	Cross-sectional survey	222	Age: younger vs older	Complete dentures	NR	None	Patient satisfaction; denture quality; retention; comfort; aesthetics; OHRQoL-related factors
da Conceição Araújo et al., [19] (2018)	Brazil	Observational longitudinal study	233	Sex: Both genders included	Maxillary and/or mandibular CDs	1 year and 5 years post-insertion	Denture use at 1 year vs 5 years; quality and satisfaction levels	Denture use, denture quality, denture integrity, user satisfaction

Comparative Clinical and Patient-Reported Outcomes

The comparative clinical and patient-reported outcomes across the included studies are detailed in Table 2. A key clinical outcome, implant survival rate, was reported as consistently high for fixed prosthetic modalities. Studies on full-arch implant-supported prostheses reported survival rates ranging from 96.2% to 100% over follow-up periods of up to six years [13-15].

**Table 2:** Comparative Clinical and Patient-Reported Outcomes of Fixed Versus Removable Prosthetic Modalities NR: not reported; 3D: three-dimensional; OHRQoL: oral health–related quality of life; OHIP-14: 14-item oral health impact profile; CD: complete denture; OD: overdenture; FP: fixed prosthesis; Mand: mandible; Max: maxilla; QoL: quality of life; N/A: not applicable; vs: versus.

Author (Year)	Prosthetic Modality	Implant Survival Rate (%)	Masticatory Efficiency	Oral Health–Related Quality of Life (OHRQoL)	Complications Reported	Key Findings
Ali et al., [10] (2025)	Conventional vs 3D-printed mandibular overdenture	NR	3D-printed > conventional	NR	NR	3D-printed showed higher occlusal force distribution but greater occlusal wear; conventional had lower wear but less force distribution
Yan et al., [11] (2025)	Implant-supported fixed prosthesis	NR	NR	NR	NR	Incisal position mainly influenced by residual ridge width, bone height, and lip length; age affected position via bone prominence; sex and lip thickness not significant
Elboraey et al., [12] (2024)	Fixed implant-retained vs removable mandibular implant-supported overdenture	NR	Improved in both; significantly higher with fixed	Improved in both; fixed superior in functional limitations domain	NR	Both modalities improved occlusal equilibration, muscle activity, and OHRQoL; fixed prosthesis demonstrated superior clinical and patient-reported outcomes
Nagni et al., [13] (2023)	Fixed full-arch implant prosthesis	98.09	Improved	NR	Joint clicks, masseter pain, prosthetic fracture	High implant survival; effective functional and aesthetic rehabilitation
Sánchez-Torres et al., [14] (2021)	Fixed complete-arch implant-supported prosthesis	100	NR	No impact (OHIP-14 ≈ 0)	Screw loosening; veneering chipping/fracture	Complications common but did not affect satisfaction or QoL
Di et al., [15] (2013)	Fixed full-arch implant prosthesis	96.2% (Mand: 99.0%; Max: 92.8%)	NR	NR	Angulated abutments; limited surgical guide use	Predictable outcomes; high implant survival
Martín‐Ares et al., [16] (2016)	CD vs OD vs FP	NR	NR	Implant-supported > CD	NR	Satisfaction varied by modality; FP showed best function, OD better hygiene, both superior to CD
Yamaga et al., [17] (2019)	Complete Dentures	NR	NR	Improved with longer use	NR	Denture use duration and quality significantly affected OHRQoL
Čelebić et al., [18] (2003)	NR	Cross-sectional survey	Complete dentures	NR	None	Patient satisfaction; denture quality; retention; comfort; aesthetics; OHRQoL-related factors
da Conceição Araújo et al. [19] (2018)	CD	N/A	NR	NR	NR	Quality and satisfaction predict long-term use

Regarding masticatory function and biomechanical performance, studies indicated advantages for fixed prostheses (FPs). Elboraey et al. [12] found that while both fixed and removable implant-supported mandibular prostheses improved occlusal equilibration and muscle activity, the fixed modality demonstrated significantly higher masticatory efficiency. Ali et al. [10] reported that 3D-printed mandibular ODs offered higher occlusal force distribution than conventional ones, though with greater occlusal wear, highlighting a trade-off within removable modalities themselves. Anatomical factors were also significant; Yan et al. [11] identified residual ridge width, bone height, and lip length as primary determinants of incisal position in fixed maxillary prostheses, with age exerting an influence through bone prominence.

Patient-reported outcomes, particularly OHRQoL, generally favored implant-supported options over conventional dentures. Multiple studies found that implant-supported prostheses, whether fixed or removable, led to significant improvements in OHRQoL [12,16,17]. Martín-Ares et al. [16] reported that patient satisfaction varied by modality, with FP offering the best functional outcomes and OD allowing for better hygiene maintenance, both being superior to CD. Similarly, Čelebić et al. [18] and da Conceição Araújo et al. [19] identified denture quality, retention, comfort, and aesthetics as critical factors linked to patient satisfaction and long-term use of conventional dentures. Notably, Sánchez-Torres et al. [14] found that despite common mechanical complications like screw loosening and veneer chipping in fixed complete-arch restorations, patient satisfaction and OHRQoL (measured by OHIP-14) remained high and were not adversely impacted.

Complications were reported across modalities but differed in nature. For fixed implant prostheses, common issues included prosthetic fractures, screw loosening, and veneering material chipping [13,14]. Studies on ODs highlighted occlusal wear as a concern [10]. Patient management factors were also evident; Nagni et al. [13] reported complications such as joint clicks and masseter pain in some patients with fixed full-arch prostheses, indicating the need for comprehensive occlusal management.

Risk of Bias Assessment

The methodological quality and risk of bias of the 10 included studies were systematically evaluated using design-specific tools. For the two RCTs [10,12], the Cochrane Risk of Bias 2 (RoB 2) tool was applied. The study by Elboraey et al. [12] was judged to have a low risk of bias across all domains. The study by Ali et al. [10] was rated as having some concerns, primarily due to the impossibility of blinding participants and personnel to the manufacturing technique (conventional versus 3D-printed), which introduced a risk of performance bias, although the objective nature of their primary outcomes (occlusal force and wear) mitigated this concern (Table 3).

**Table 3:** Risk of Bias Assessment for Randomized Controlled Trials (Using the RoB 2 Tool) RoB: Risk of Bias 2

Study (Year)	Randomization Process	Deviations from Intended Interventions	Missing Outcome Data	Measurement of the Outcome	Selection of the Reported Result	Overall Bias Judgement
Ali et al., [10] (2025)	Low	Some concerns	Low	Low	Low	Some concerns
Elboraey et al., [12] (2024)	Low	Low	Low	Low	Low	Low

For the remaining cohort, observational, and cross-sectional studies [11,13-19], the NOS was utilized. All eight studies were assessed as good quality, with scores ranging from 6 to 8 stars, as detailed in Table 4. This indicates a generally low risk of bias within the inherent constraints of non-randomized designs. Common strengths across these studies included representative patient samples and the use of validated outcome measures, such as the Oral Health Impact Profile (OHIP) for assessing quality of life [14,16,17]. The primary limitations stem from the observational nature of the evidence, including the potential for residual confounding (e.g., baseline bone quality, surgical skill) and selection bias in retrospective analyses [11,13-15]. Despite these limitations, the consistent direction of findings across studies of varying designs and geographical contexts strengthens the credibility of the synthesized evidence (Table 4).

**Table 4:** Risk of Bias Assessment for Cohort and Observational Studies (Using the Newcastle-Ottawa Scale). Stars (★) represent the number of points awarded in each domain: Selection (maximum 4 stars), Comparability (maximum 2 stars), and Outcome (maximum 3 stars). Studies scoring 6–8 stars were considered to have good methodological quality.

Study (Year)	Selection (Max 4)	Comparability (Max 2)	Outcome (Max 3)	Total Stars	Quality
Yan et al., [11] (2025)	★★★	★★	★★	7	Good
Nagni et al., [13] (2023)	★★★	★	★★	6	Good
Sánchez-Torres et al., [14] (2021)	★★★★	★★	★★	8	Good
Di et al., [15] (2013)	★★	★★	★★	6	Good
Martín‐Ares et al., [16] (2016)	★★★★	★★	★★	8	Good
Yamaga et al., [17] (2019)	★★★★	★★	★★	8	Good
Čelebić et al., [18] (2003)	★★★★	★★	★★	8	Good
da Conceição Araújo et al., [19] (2018)	★★★	★★	★★	7	Good

Discussion

This systematic review synthesized evidence from 10 studies to evaluate the comparative clinical and patient-reported outcomes of fixed versus removable prosthetic modalities for completely edentulous patients. The findings present a nuanced picture, indicating that while implant-supported prostheses, both fixed and removable, offer substantial advantages over conventional CDs, the choice between an FP and a removable OD involves a critical trade-off between biomechanical performance, quality of life, and clinical manageability. The analysis reveals consistently high implant survival rates for fixed modalities, superior masticatory function with FPs, and significant improvements in OHRQoL with any form of implant therapy. However, these benefits are tempered by modality-specific complications and anatomical and patient-specific factors that must guide clinical decision-making.

The cornerstone of implant therapy’s success is long-term survival, and our review affirms the predictability of fixed full-arch reconstructions. Studies by Di et al. [15], Nagni et al. [13], and Sánchez-Torres et al. [14] reported survival rates between 96.2% and 100% over follow-up periods extending to six years. These findings align robustly with the established literature on full-arch protocols like All-on-4®. A 2022 meta-analysis by Papaspyridakos et al. [20] similarly reported a 10-year implant survival rate of 96.7% for fixed complete dental prostheses, underscoring the procedural reliability when executed with precision. Our included studies, particularly that by Di et al. [15], who reported a lower survival rate in the maxilla (92.8%) compared to the mandible (99.0%), also echo a well-documented trend in implant dentistry. This discrepancy is frequently attributed to the generally poorer bone quality and the anatomical challenges of the maxilla, a factor extensively discussed in a systematic review by Chrcanovic et al. [21], which identified the jaw as a significant implant failure risk factor. Therefore, while overall survival is high, our review reinforces that treatment planning must account for anatomical site-specific risks.

Beyond survival, the restoration of function is a primary goal. Our synthesis indicates a clear biomechanical advantage for FPs. Elboraey et al. [12] provided direct comparative evidence, demonstrating that while both FPs and ODs improved occlusal equilibration and muscle activity, the fixed modality yielded significantly higher masticatory efficiency. This finding is physiologically coherent, as a fixed prosthesis provides a more stable and rigid platform for mastication, closely mimicking the natural dentition. This advantage is further supported by the study of Ali et al. [10], which, while comparing fabrication techniques for ODs, indirectly highlighted the functional limitations inherent to removable designs; even the superior 3D-printed OD exhibited concerning occlusal wear. The biomechanical superiority of FPs is consistent with earlier comparative studies. For instance, a systematic review by Emami et al. [22] concluded that implant-supported FPs offer better chewing ability and patient-reported masticatory performance than implant ODs. However, our review introduces an important caveat from Yan et al. [11], who emphasized that achieving an optimal and biomechanically sound incisal position for an FP is heavily dependent on residual bone dimensions and facial morphology. This underscores that the functional superiority of an FP is contingent upon meticulous three-dimensional treatment planning, as suboptimal implant positioning driven by anatomical constraints can compromise the final prosthetic outcome, a point strongly advocated in the work of Buser et al. [23] on criteria for ideal implant placement.

Perhaps the most compelling argument for implant therapy, regardless of the chosen modality, is its profound impact on patient-reported outcomes, particularly OHRQoL. The evidence synthesized here is unequivocal: implant-supported prostheses dramatically enhance quality of life compared to conventional dentures. Martín-Ares et al. [16] found that both FPs and ODs were superior to CDs, with FPs rated highest for function and ODs for ease of hygiene. This patient-perceived hierarchy is crucial for shared decision-making. Importantly, Sánchez-Torres et al. [14] made a critical observation that mechanical complications (e.g., screw loosening, veneer chipping), while common, did not negatively impact patient satisfaction or OHRQoL scores. This suggests that patients value the stability and security of a fixed restoration so highly that they tolerate a certain level of maintenance, a sentiment reflected in longitudinal studies by Bozini et al. [24], who found high patient satisfaction with implant prostheses despite reported technical issues. For conventional denture wearers, factors influencing satisfaction are more fundamental. Studies by Čelebić et al. [18] and da Conceição Araújo et al. [19] identified denture quality, retention, comfort, and aesthetics as the primary drivers of OHRQoL and long-term use. The finding by Yamaga et al. [17] that OHRQoL improves with longer denture use period likely reflects patient adaptation and the progressive refinement of the neuromuscular denture skill.

The review also elucidates a distinct profile of complications for each modality, informing long-term maintenance expectations. For fixed implant prostheses, the issues are predominantly technical and biological in nature. Sánchez-Torres et al. [14] and Nagni et al. [13] reported complications such as prosthetic fracture, screw loosening, and veneer chipping. These findings are highly consistent with a large systematic review by Pjetursson et al. [25], which identified technical complications as the most frequent reason for intervention in implant-supported fixed reconstructions over a 10-year period. Furthermore, Nagni et al. [13] reported biological complications like peri-implantitis and, notably, instances of joint clicks and masseter pain. This highlights the critical importance of achieving a harmonious occlusion in a rigid, cross-arch splinted prosthesis to avoid temporomandibular dysfunction, a risk factor discussed by Kim et al. in the context of full-arch rehabilitations. For removable ODs, the complications shift towards prosthetic wear and maintenance, as seen in the study by Ali et al. [10]. CDs, as implied by the studies in this review [18,19], face challenges primarily related to retention, stability, and mucosal health, leading to a higher rate of dissatisfaction and disuse, a well-documented problem in edentulous populations.

When interpreting these findings, the methodological quality of the evidence must be considered. The risk of bias assessment presents a mixed picture. The two RCTs [10,12] provide the highest level of evidence for direct comparisons, with Elboraey et al. [12] judged as low risk. However, the preponderance of evidence comes from observational studies [11,13-19], all rated as good quality but inherently limited by potential confounding and selection bias. For example, in retrospective studies by Yan et al. [11] and Nagni et al. [13], the outcomes may be influenced by unmeasured variables such as surgical expertise or pre-operative bone quality, which are not randomized. This is a common limitation in surgical prosthetic research, where long-term RCTs are challenging to conduct. Nevertheless, the convergence of findings across different study designs, populations, and geographical locations strengthens the overall conclusions. The observed patterns-high implant survival, functional superiority of FPs, major QoL gains with implants, and modality-specific complications-are corroborated by broader systematic reviews. For example, our finding that OHRQoL improves with implant support is strongly supported by the Cochrane review by Thomason et al., which concluded that implant ODs in the mandible are more effective than conventional dentures in improving patient satisfaction and OHRQoL. Similarly, the trade-off between function and hygiene between FPs and ODs has been a consistent theme in the literature, as noted in a review by Feine et al. [26], who advocated for the mandibular two-implant OD as a first-choice standard of care due to its optimal balance of benefits, costs, and risks. Our review suggests that while this remains a valid standard, for patients where biomechanical performance is the absolute priority and who are willing to accept more complex hygiene routines, an FP may be the preferred option.

Limitations

This systematic review has several limitations. First, the included studies are heterogeneous in their designs, populations, prosthetic protocols (e.g., number of implants, loading protocols), and outcome measures, precluding a formal meta-analysis and demanding a narrative synthesis. Second, the follow-up durations vary widely, from 3 months to 6 years, limiting the ability to draw firm conclusions about very long-term outcomes. Third, while the NOS ratings were good, the observational nature of most studies means that the evidence is susceptible to confounding by indication; for instance, patients receiving FPs may have had better baseline bone volume or higher socioeconomic status, potentially inflating the observed advantages. Fourth, the review primarily captures patient-reported outcomes through generic OHRQoL instruments like OHIP; condition-specific or prosthesis-specific satisfaction measures might provide deeper insights. Finally, economic evaluations and cost-effectiveness analyses were outside the scope of this review, yet these are decisive factors in real-world clinical decision-making for many patients and healthcare systems.

## Conclusions

This systematic review confirms that for completely edentulous patients, implant-supported prosthetic modalities-both fixed and removable-offer substantially superior clinical and patient-reported outcomes compared to conventional complete dentures. The choice between a fixed prosthesis and a removable overdenture is not one of superior versus inferior, but rather a strategic decision based on a hierarchy of patient priorities. Fixed prostheses provide the highest level of masticatory function, stability, and often patient-perceived quality of life, but require acceptance of more complex hygiene procedures and a higher likelihood of technical complications requiring intervention. Removable overdentures offer an excellent compromise, with vastly improved function and quality of life over conventional dentures, easier cleaning access, and generally lower technical complexity and cost, albeit with potentially more prosthetic maintenance like relining or attachment replacement. The decision must be guided by a thorough assessment of the patient’s anatomical realities, functional demands, hygiene ability, aesthetic expectations, and financial considerations. Ultimately, the optimal treatment is the one that aligns with the individual patient’s values and circumstances, facilitated by a clinician skilled in both modalities. Future research should prioritize long-term, pragmatic RCTs directly comparing these modalities and incorporating comprehensive economic analyses to further refine evidence-based clinical guidelines.

## Appendices

**Table 5:** Search Strings for all Databases

Database	Full Search String
PubMed (MEDLINE)	(("Edentulous"[Mesh] OR edentulous[tiab] OR "complete edentulism"[tiab] OR "completely edentulous"[tiab]) AND ("Dental Prosthesis, Implant-Supported"[Mesh] OR "implant-supported"[tiab] OR "fixed dental prosthesis"[tiab] OR "fixed complete denture"[tiab] OR "fixed prosthesis"[tiab] OR "implant-supported fixed"[tiab]) AND ("Dental Prosthesis"[Mesh] OR "complete denture"[tiab] OR overdenture*[tiab] OR "removable dental prosthesis"[tiab] OR "implant-retained overdenture"[tiab] OR "removable prosthesis"[tiab]) AND (outcome*[tiab] OR survival[tiab] OR success[tiab] OR complication*[tiab] OR "patient satisfaction"[tiab] OR "quality of life"[tiab] OR masticatory[tiab]))
Scopus	(TITLE-ABS-KEY (edentulous OR "complete edentulism" OR "completely edentulous") AND TITLE-ABS-KEY ("implant-supported" OR "fixed dental prosthesis" OR "fixed complete denture" OR "fixed prosthesis") AND TITLE-ABS-KEY ("complete denture" OR overdenture* OR "removable dental prosthesis" OR "implant-retained overdenture") AND TITLE-ABS-KEY (outcome* OR survival OR success OR complication* OR "patient satisfaction" OR "quality of life" OR masticatory))
Web of Science (Core Collection)	TS=(edentulous OR "complete edentulism" OR "completely edentulous") AND TS=("implant-supported" OR "fixed dental prosthesis" OR "fixed complete denture" OR "fixed prosthesis") AND TS=("complete denture" OR overdenture* OR "removable dental prosthesis" OR "implant-retained overdenture") AND TS=(outcome* OR survival OR success OR complication* OR "patient satisfaction" OR "quality of life" OR masticatory)
Embase	('edentulous'/exp OR edentulous:ti,ab OR 'complete edentulism':ti,ab) AND ('implant supported dental prosthesis'/exp OR 'fixed dental prosthesis':ti,ab OR 'implant supported':ti,ab OR 'fixed complete denture':ti,ab) AND ('complete denture'/exp OR overdenture*:ti,ab OR 'removable dental prosthesis':ti,ab OR 'implant retained overdenture':ti,ab) AND (outcome*:ti,ab OR survival:ti,ab OR success:ti,ab OR complication*:ti,ab OR 'patient satisfaction':ti,ab OR 'quality of life':ti,ab OR masticatory:ti,ab)
